# Myocardial Energetics in Obesity: Enhanced ATP Delivery Through Creatine Kinase With Blunted Stress Response

**DOI:** 10.1161/CIRCULATIONAHA.119.042770

**Published:** 2020-04-06

**Authors:** Jennifer J. Rayner, Mark A. Peterzan, William D. Watson, William T. Clarke, Stefan Neubauer, Christopher T. Rodgers, Oliver J. Rider

**Affiliations:** 1 Oxford Centre for Clinical Magnetic Resonance Research, Division of Cardiovascular Medicine, Radcliffe Department of Medicine (J.J.R, M.A.P., W.D.W., S.N., O.J.R.), University of Oxford, John Radcliffe Hospital, United Kingdom.; 2 Wellcome Centre for Integrative Neuroimaging, Oxford Centre for Functional MRI of the Brain (W.T.C.), University of Oxford, John Radcliffe Hospital, United Kingdom.; 3 Wolfson Brain Imaging Centre, University of Cambridge, Cambridge Biomedical Campus, United Kingdom (C.T.R.).

**Keywords:** heart failure, magnetic resonance spectroscopy, obesity

## Abstract

**Background:**

Obesity is strongly associated with exercise intolerance and the development of heart failure. Whereas myocardial energetics and diastolic function are impaired in obesity, systolic function is usually preserved. This suggests that the rate of ATP delivery is maintained, but this has never been explored in human obesity. We hypothesized that ATP transfer rate through creatine kinase (CK) (*k*_f_^CKrest^) would be increased, compensating for depleted energy stores (phosphocreatine/ATP), but potentially limiting greater ATP delivery during increased workload. We hypothesized that these changes would normalize with weight loss.

**Methods:**

We recruited 80 volunteers (35 controls [body mass index 24±3 kg/m^2^], 45 obese [body mass index 35±5 kg/m^2^]) without coexisting cardiovascular disease. Participants underwent body composition analysis, magnetic resonance imaging of abdominal, liver, and myocardial fat content, left ventricular function, and ^31^P magnetic resonance spectroscopy to assess phosphocreatine/ATP and CK kinetics, at rest and during dobutamine stress. Obese volunteers were assigned to a dietary weight loss intervention, before reexamination.

**Results:**

At rest, although myocardial phosphocreatine/ATP was 14% lower in obesity (1.9±0.3 versus 2.2±0.2, *P*<0.001), *k*_f_^Ckrest^ was 33% higher (0.23±0.07 s^–1^ versus 0.16±0.08 s^–1^, *P*=0.002), yielding no difference in overall resting ATP delivery (obese 2.5±0.9 µmol·g^–1^·s^–1^ versus control 2.2±1.1 µmol·g^–1^·s^–1^, *P*=0.232). In controls, increasing cardiac workload led to an increase in both *k*_f_^CK^ (+86%, *P*<0.001) and ATP delivery (+80%, *P*<0.001). However, in obesity, similar stress led to no significant increase in either *k*_f_^CK^ (*P*=0.117) or ATP delivery (*P*=0.608). This was accompanied by reduced systolic augmentation (absolute increase in left ventricular ejection fraction, obese +16±7% versus control +21±4%, *P*=0.031). Successful weight loss (–11±5% body weight) was associated with improvement of these energetic changes such that there was no significant difference in comparison with controls.

**Conclusions:**

In the obese resting heart, the myocardial CK reaction rate is increased, maintaining ATP delivery despite reduced phosphocreatine/ATP. During increased workload, although the nonobese heart increases ATP delivery through CK, the obese heart does not; this is associated with reduced systolic augmentation and exercise tolerance. Weight loss reverses these energetic changes. This highlights myocardial energy delivery through CK as a potential therapeutic target to improve symptoms in obesity-related heart disease, and a fascinating modifiable pathway involved in the progression to heart failure, as well.

## Abstract 2

Supplemental Digital Content is available in the text.

**Clinical Perspective**

**What Is New?**

- Obesity is normally associated with depleted myocardial energetics, and yet here we demonstrate that energy delivery may be maintained at rest through increased work through the creatine kinase shuttle.
- Although this maintains energy delivery at rest, during stress the system does not increase delivery rate further, which is linked to limitation on cardiopulmonary exercise testing.
- These energetic changes seem to be reversed with intentional weight loss, alongside structural cardiac remodeling.

**What Are the Clinical Implications?**

- Exertional symptoms are highly prevalent in obesity, and these data may represent a cardiac explanation for this limitation.
- Obesity is also one of the strongest risk factors for the development of heart failure with preserved ejection fraction, where a similar loss of myocardial reserve is seen. It would be interesting to see whether a similar loss of energetic flexibility is common to both conditions.
- This pathway may represent a therapeutic target in obesity cardiomyopathy and potentially in heart failure with preserved ejection fraction, aimed at restoring energetic capacity to cope with increased stress.

**Editorial, see p 1164**

Obesity is the most significant health issue in modern society, and levels continue to rise rapidly.^1^ It is associated with the entire range of cardiac functional changes, from subclinical diastolic dysfunction^2^ to overt heart failure.^3^ To maintain normal contraction and relaxation, the myocardium requires a constant supply of ATP. Increased adiposity is known to be associated with a wide range of hemodynamic and metabolic shifts that increase myocardial ATP demand. A mismatch in myocardial ATP supply and demand could represent a unifying pathophysiological mechanism linking obesity and myocardial dysfunction.

The creatine kinase (CK) shuttle is thought to be the main transfer mechanism involved in transferring ATP from the mitochondrion to the myofibril, using the rapid diffusion capability of phosphocreatine (PCr) to transfer a phosphate group onto ATP at the myofibril. This equilibrium reaction is catalyzed by CK: PCr+ADP⇌creatine+ATP. The forward rate constant of the myocardial CK reaction is termed *k*_f_^CK^, and ATP delivery to the myocardium can be calculated as *k*_f_^CK^×[PCr].^4^ A reduction in ATP delivery through this pathway has been shown to be associated with decompensation toward heart failure in the pressure-loaded heart,^5^ and worsening prognosis in established heart failure, as well.^6^

Although, in obesity, the availability of PCr (or PCr pool) is reduced, with a lower PCr/ATP ratio on ^31^P magnetic resonance (MR) spectroscopy^2^ similar to that recorded in heart failure with reduced ejection fraction,^7^ systolic function is typically preserved. We hypothesized that in obesity with normal systolic function, the forward rate constant through CK would be elevated, thus maintaining ATP delivery in the face of PCr depletion. If this were the case, the demand incurred by increasing myocardial workload may outstrip ATP supply, unmasking functional impairment and symptoms even in those who have normal resting cardiac function.

Therefore, we used the combination of advanced ^31^P MR spectroscopy and cardiac MR imaging, to investigate myocardial CK kinetics, PCr/ATP and cardiac function at rest and during catecholamine stress in obesity, and compared this with the nonobese heart. To assess any potential reversibility, we also investigated the effect of intentional weight loss on these measures.

## Methods

The study was approved by the local ethics board (National Research Ethics Service reference 14/SC/004), and was in accordance with the Declaration of Helsinki. Eighty volunteers were recruited from the Oxfordshire community in the United Kingdom, comprising 45 obese (body mass index [BMI] >30 kg/m^2^) and 35 nonobese controls (BMI <30 kg/m^2^). All signed written consent for the study investigations. There were no race- or sex-based differences between study groups. All primary data are available on reasonable request from the corresponding author. There is no scientific overlap with previous studies by this group.^2^

### Exclusion Criteria

Participants were excluded if they had any history of heart failure, coronary artery disease, more than mild valvular disease, uncontrolled hypertension (defined as resting blood pressure >180/90) or atrial fibrillation (heart rate >110 bpm), or diabetes mellitus (fasting glucose >7.1 mmol/L), as was any contraindication to MR scanning.

### Anthropomorphic and Biochemical Assessment

Height, weight, and body composition were measured using digital scales with bioimpedance analysis (InBody 770, InBody Co Ltd). Body surface area was calculated using the Mosteller formula (body surface area [m^2^]=√[ height×weight]/3600). Noninvasive blood pressure was measured according to standardized methods (average of 3 supine measurements with an automatic sphygmomanometer, Carescape V100, GE). Fasting venous blood was drawn and biomarkers were analyzed either by the Oxford University Hospitals clinical biochemistry laboratory according to standardized protocols, or by commercially available enzyme-linked immunosorbent assay kit (leptin; Sigma-Aldrich). Fasting insulin resistance was represented by homeostatic model assessment of insulin resistance ([glucose×insulin]/22.5).

### MR Imaging

All MR imaging and spectroscopy was acquired on a 3T MR system (Tim Trio, Siemens). Abdominal visceral fat volume was measured with a 5-mm transverse slice at the level of the 5th lumbar vertebral body, using a water-suppressed turbo spin echo sequence and analyzed with manual contouring as previously described.^8^ Liver fat was calculated using the Dixon method, with analysis using Liver MultiScan (Perspectum Diagnostics).^9^ Myocardial triglyceride concentration was quantified with 1H spectroscopy, using a single-voxel stimulated echo acquisition mode sequence as previously described.^10^

Cardiac imaging to quantify ventricular volumes and function was acquired using a steady-state free precession sequence (echo time, 1.5 ms; repetition time, 3 ms), which was performed with cardiac triggering and during end-expiratory breath-hold. Endocardial and epicardial left ventricular contours were drawn manually and analyzed using a semiautomated system (cmr42, Circle Cardiovascular Imaging Inc). Left ventricular stroke work was calculated as stroke volume×mean arterial pressure. Rate pressure product was calculated as heart rate×systolic blood pressure.

Cardiac MR imaging data were analyzed by an experienced observer, blinded to baseline group, time point in the obese cohort, and whether the weight loss intervention was successful.

### ^31^P MR Spectroscopy

^31^P MR spectroscopy was performed on the same 3T system described earlier, with a 10-cm loop transmit-receive 31P surface coil (Pulse Teq). The CK forward rate constant *k*_f_ was measured using a modified one-dimensional chemical shift imaging Triple Repetition Saturation Transfer (TriST)^11^ sequence with a shorter stressed saturation transfer extension.^12^ TriST measures 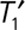, 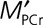 and 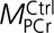 This requires 3 steps: 2 to measure 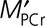 and 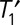 using the dual-repetition time method, and a third to measure 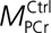. The pseudo–first-order forward rate constant (*k*_f_) of CK was then calculated according to the following formula:


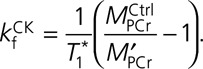


*T*_1_* is a hypothetical longitudinal relaxation constant for a molecule without chemical exchange. Assuming that *T*_1_* does not change from one scan to the next (eg, in myocardium at rest versus stress), then an additional measurement of CK *k*_f_ may be made in 2 steps: measuring only 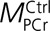 and 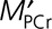 The stressed saturation transfer protocol comprises a rest measurement of CK *k*_f_ and PCr *T*_1_* using TriST (4 steps) and a measurement of CK *k*_f_ during intravenous dobutamine stress (2 additional steps described earlier to calculate the CK *k*_f_ during increased work load). The 6 (4+2) steps were completed in a single scanning session. The relative ratio of PCr to γ-ATP peaks in the fully relaxed spectrum was used to generate a PCr/ATP ratio, and the rate of ATP delivery, or CK flux was calculated by *k*_f_×[PCr], where [PCr] is calculated by multiplying PCr/ATP by literature values for [ATP].^4^ It was assumed that myocardial [ATP] is similar in obesity in the absence of systolic dysfunction as it is in controls, as observed in animal models.^13^ Because measurements of CK *k*_f_ were acquired supine, an adjustment factor of 1.333 was applied to align values with the published normal range (acquired prone)^12^; hence, all *k*_f_ values reported throughout the article have been adjusted using this scaling factor.

### Dobutamine Stress Measurements

A subset of volunteers (n=20) prespecified by the Ethics Committee underwent dobutamine stress. Any β-blocker therapy was withheld for a period of 48 hours before the study visit. Dobutamine was infused through a peripheral venous cannula, at incremental doses between 5 and 40 µg·kg^–1^·min^–1^ as necessary to achieve the target heart rate of 65% maximum (maximum heart rate calculated as 220 – age). Cine images were repeated at maximum stress to assess contractile response to stress, and exclude any regional wall motion abnormalities, as well. 31P MR spectroscopy data were analyzed post hoc using AMARES analysis software within MATLAB as previously described^12,14^ and therefore not at risk of interpretation bias. Average time of infusion was 24 minutes.

### Echocardiography

Echocardiography was performed on a Philips Epiq system to determine diastolic function; pulse wave velocity was measured at mitral valve inflow to calculate E/A ratio, and tissue Doppler at lateral and medial mitral valve annulus to generate E/e′ ratios, and the mean of medial and lateral velocities, as well.

### Cardiopulmonary Exercise Testing

All participants underwent maximal exercise testing on a cycle ergometer according to an individualized ramped protocol. Continuous recording of respiratory gas exchange parameters was taken during the test and recovery (Cortex Metalyser). Gas exchange data were acquired breath by breath and averaged over 20-second intervals. Heart rate reserve was calculated as increase in heart rate (HR) with exercise divided by difference between resting HR and age-predicted maximum HR.^15^

### Six-Minute Walk Test

All participants underwent a standardized walking test^16^ along a 35-m corridor for 6 minutes. The total distance achieved was recorded.

### Weight Loss Intervention

The obese volunteers underwent dietary weight loss advice with telephone/email support from the study team. They adhered to a calorie-controlled (up to 1500 kcal), low glycemic index diet for a period of 6 months (average, 194 days; interquartile range, 78–361 days). Volunteers were encouraged to maintain current activity levels during this timeframe.

### Statistical Analysis

Statistical analysis was performed using commercial software (SPSS 24). All data are presented as mean±SD unless stated otherwise. Equality of variance was evaluated using the Levene test, before determination of statistical significance, either by χ^2^ tests in the case of categorical data, or Student *t* tests for continuous data (paired or independent where relevant). Pearson correlation and linear regression were used. Values of *P*<0.05 were considered as statistically significant.

## Results

### Anthropometric Data

**Figure 1.**
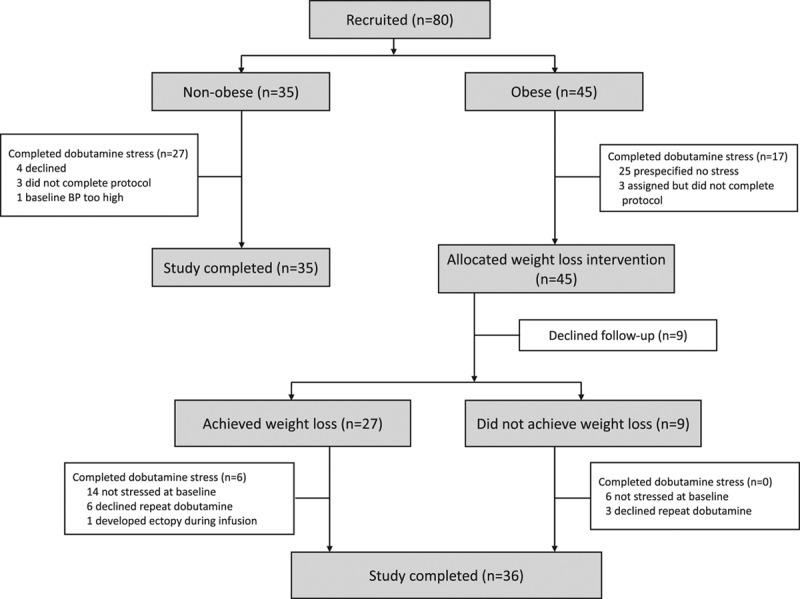
**Study design.** CONSORT diagram (Consolidated Standards for Reporting of Trials) illustrating volunteer pathway through study investigations.BP indicates blood pressure.

When comparing groups at baseline (n=45 obese, n=35 nonobese controls; Figure 1 and Table 1) as expected, obese volunteers had significantly higher BMI (35±5 kg/m^2^ versus 24±3 kg/m^2^, *P*<0.001) and total body fat mass (44±11 kg versus 16±7 kg, *P*<0.001). Abdominal visceral fat was elevated in comparison with controls (156±81 cm^2^ versus 49±42 cm^2^, *P*<0.001). The 2 groups were well-balanced for age and sex. Blood pressure was significantly higher in the obese group (systolic 136±19 mm Hg versus 126±19 mm Hg in controls, *P*=0.029; diastolic 85±12 versus 73±13, *P*<0.001) despite 3 of 47 obese volunteers taking a single antihypertensive agent, as was resting heart rate (67±13 bpm versus 57±8 bpm, *P*=0.001). Total cholesterol, triglycerides, glucose, insulin, and leptin were all significantly higher in the obese group (Table 1). Of the 45 obese volunteers, 27 (60%) would be defined as insulin resistant (homeostatic model assessment of insulin resistance>2.0^17^), and 18 (40%) fulfilled the criteria for a diagnosis of nonalcoholic fatty liver disease (>5% liver fat). Baseline brain natriuretic peptide readings did not significantly differ (nonobese 7±5 mmol/L versus obese 5±5 mmol/L, *P*=0.183).

**Table 1.**
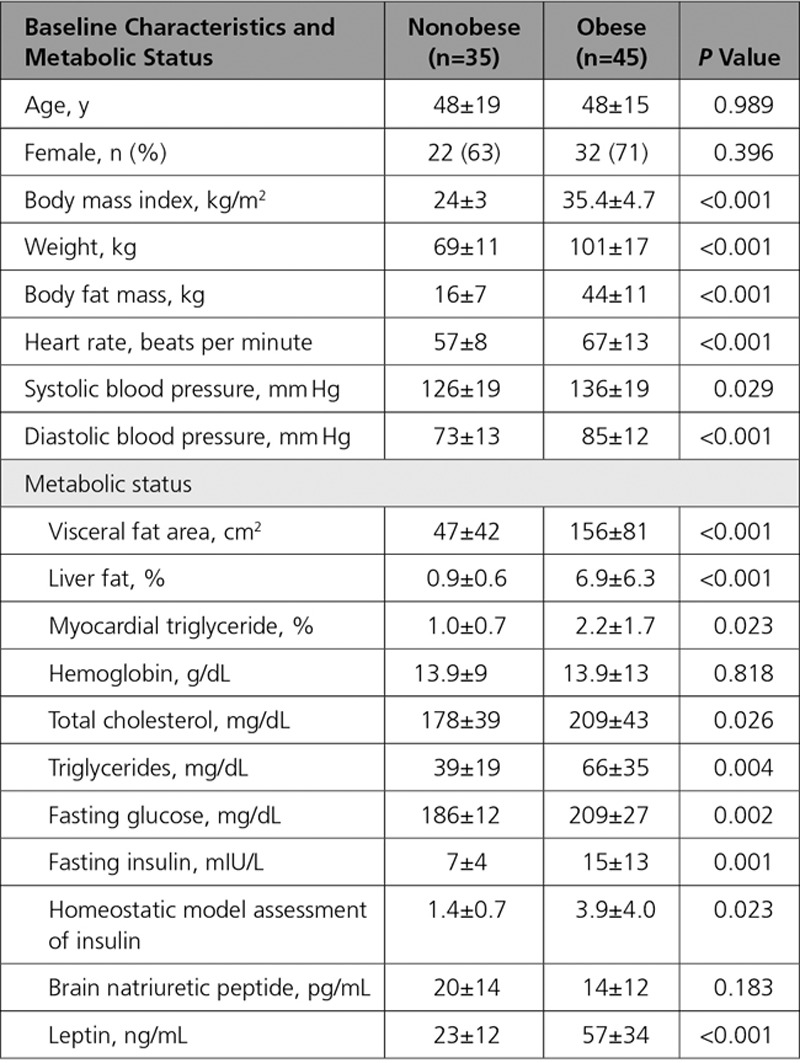
Baseline Anthropomorphic and Metabolic Findings

### Impact of Obesity on Cardiac Structure and Function

In line with previous reports, obesity was associated with left ventricle (LV) remodeling with elevated LV mass (114±27 g versus 99±19 g, *P*=0.015, Table 2) and a trend toward end-diastolic volume enlargement (158±33 mL versus 149±26 mL, *P*=0.181). Systolic function was relatively hyperdynamic in the obese group, with ejection fraction 66±4% versus 63±5% in the nonobese group (*P*=0.002). There was a greater degree of diastolic dysfunction in the obese group (E/e′ 9.1±2.0 versus 7.6±1.4, *P*=0.044), with left atrial dilatation (obese group left atrium volume 78±21 mL versus nonobese 56±14 mL, *P*<0.001).

**Table 2.**
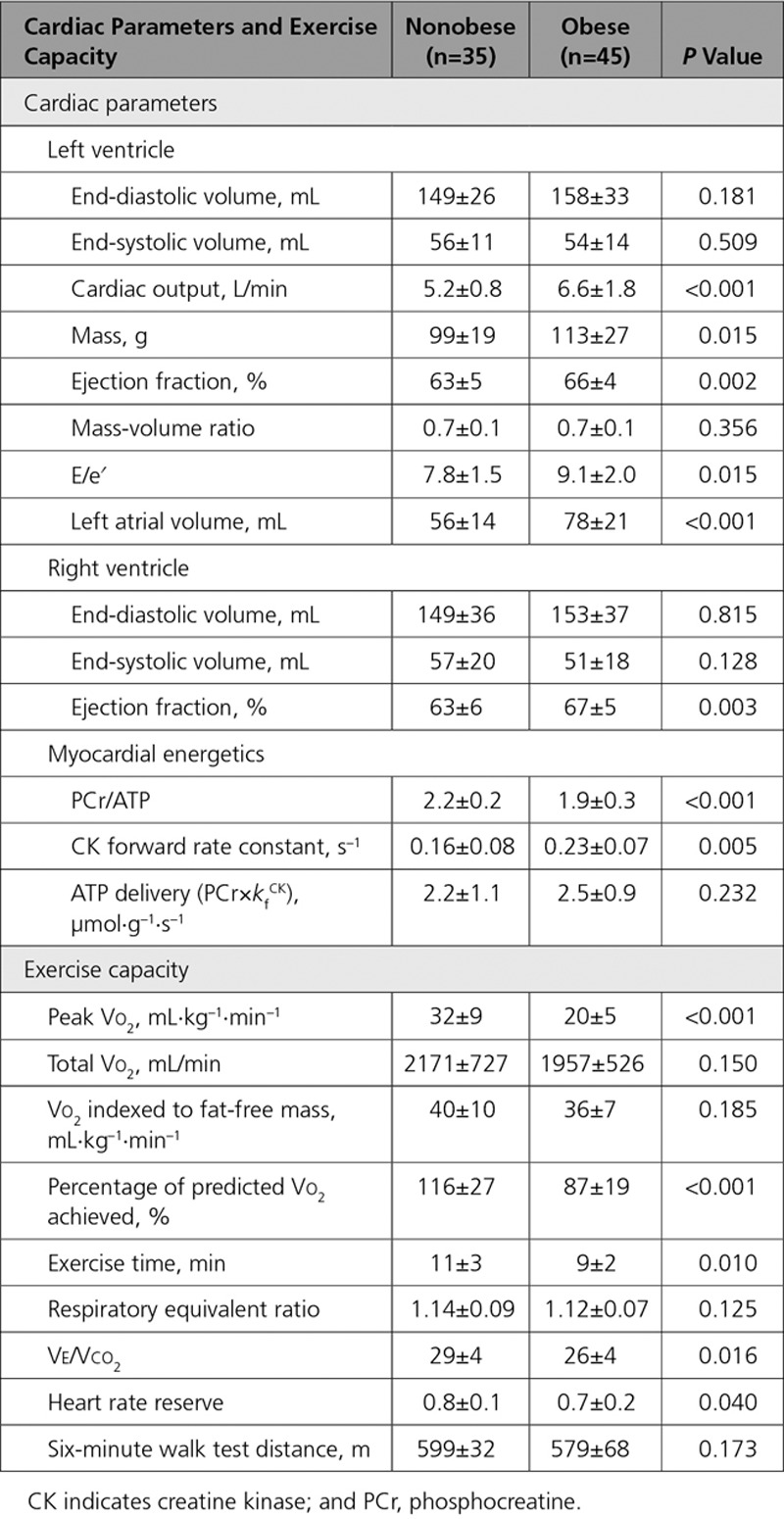
Baseline Cardiac Parameters and Exercise Capacity

### Obesity and Exercise Capacity

Participants underwent cardiopulmonary exercise testing, with all achieving adequate tests, and no difference between the groups in terms of effort (respiratory equivalent ratio, nonobese 1.14±0.09 versus obese 1.12±0.07, *P*=0.125; Table 2).

The obese group showed limitation of maximal performance, with lower peak Vo_2_ at 20±5 mL·kg^–1^·min^–1^ (in comparison with nonobese 32±9 mL·kg^–1^·min^–1^, *P*<0.001), and lower percentage of predicted Vo_2_ achieved (87±19% versus control 116±27%, *P*<0.001). When comparing the less bodyweight–dependent variables, total exercise time (obese 9±2 minutes versus control 11±3 minutes, *P*=0.010) and HR reserve (obese 0.7±0.2 versus control 0.8±0.1, *P*=0.040), these also showed decreased exercise capacity and relative chronotropic incompetence. There was, however, no difference in 6-minute walk test distance.

### Impact of Obesity on Myocardial Energetics

**Figure 2.**
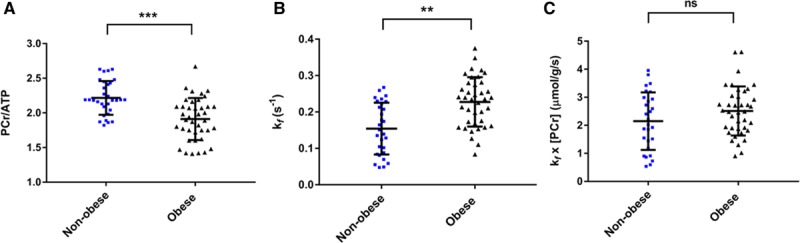
**Myocardial energetic differences in obesity.** Obesity is associated with lower PCr/ATP values (**A**), increased forward rate constant of the CK reaction (**B**), and a trend toward increased CK flux (**C**). **P<0.01; ***P<0.001 on independent *t* test. CK indicates creatine kinase; ns, not significant; and PCr, phosphocreatine.

Obesity was associated with reduced PCr/ATP (1.9±0.3 versus 2.2±0.2, *P*<0.001; Figure 2). In contrast, the forward rate constant of CK was 44% higher in the obese group than in the control group (all *k*_f_ values rescaled with adjustment factor [see Methods]; *k*_f_^Ckrest^ 0.23±0.07 s^–1^ versus 0.16±0.08 s^–1^, *P*=0.002; Figure 2). If both obese and nonobese groups are combined, *k*_f_^Ckrest^ was positively correlated with BMI (*r* 0.329, *P*=0.006), fat mass (*r* 0.393, *P*=0.005), visceral fat (*r* 0.316, *P*=0.020, Figure I in the Data Supplement), and leptin concentration (*r* 0.355, *P*=0.012), but not with age, myocardial triglyceride content, or any resting functional parameter (Table I in the Data Supplement). If *k*_f_^CKrest^ was indexed to BMI or fat mass, there was no significant difference between groups (BMI, *P*=0.404; fat mass, *P*=0.058), whereas a significant difference persisted when adjusting for visceral fat (*P*=0.008). The increased *k*_f_^CKrest^ more than compensated for the reduced PCr/ATP, with ATP delivery (calculated as [PCr]×*k*_f_^CK^) in obesity demonstrating no difference (2.5±0.9 µmol·g^–1^·s^–1^ versus 2.2±1.1 µmol·g^–1^·s^–1^, *P*=0.232).

Resting LV stroke work was higher in obesity (10.2±2.4 L×mm Hg versus 8.4±2.1 L×mm Hg, *P*=0.001). When *k*_f_^CKrest^ was indexed to either LV stroke work or rate-pressure product, there was no significant difference between control and obese groups (stroke work *P*=0.136, rate pressure product *P*=0.576). *k*_f_^CKrest^ correlated with LV stroke work (*r*=0.309, *P*=0.016) considering both groups together.

Overall, this suggests that in obesity, at rest, the required increase in LV stroke work increases ATP demand, and that, in the face of reduced PCr/ATP, the forward rate constant through CK is elevated to compensate and maintain myocardial ATP delivery.

### Effects of Catecholamine Stress

#### Hemodynamics

Fifty participants (30 nonobese and 20 obese) embarked on the dobutamine stress protocol (Figure 1); these volunteers were representative of the group as a whole (Tables II and III in the Data Supplement). This was well-tolerated, with no significant adverse effects and only 6 volunteers not completing the protocol because of either nausea (n=2) or physical discomfort from prolonged scanning (n=4); therefore, 27 nonobese and 17 obese participants completed the protocol. There was no significant difference in dobutamine doses between the 2 groups (normal weight 27±8 μg·mL^–1^·min^–1^ versus obese 25±7 μg·mL^–1^·min^–1^, *P*=0.621). Stress elicited similar hemodynamic responses in both groups, namely increases in mean HR of 65%, in mean rate pressure product of 152%, and in mean LV stroke work of 22% (see Table IV in the Data Supplement). Dobutamine led to significant increases in ejection fraction in both obese (by 16±7%, *P*<0.001 versus rest) and nonobese groups (by 21±4%, *P*<0.001 versus rest); however, the contractile response to stress in terms of relative increase in ejection fraction was greater in controls (*P*=0.023 comparing groups) reflecting greater baseline ejection fraction in the obese cohort.

#### Myocardial Energetics

**Figure 3.**
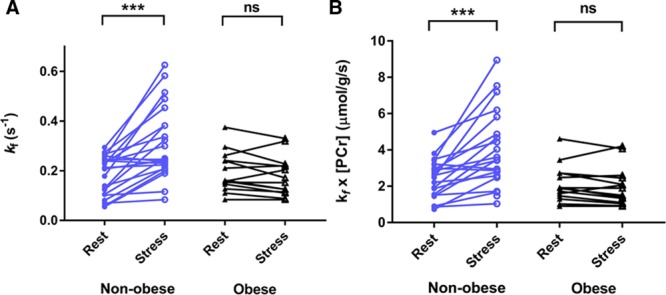
**The impact of stress on myocardial energetics.** The effects of catecholamine stress on CK *k*_f_ (**A**) and CK flux (**B**) in nonobese and obese individuals. ***P<0.001 (paired *t* test). CK indicates creatine kinase; ns, not significant; and PCr, phosphocreatine.

At this moderate level of stress, no change in PCr/ATP was seen in either group (comparing only those who underwent stress: nonobese 2.2±0.2 at rest versus stress 2.2±0.5, *P*=0.959; obese rest 1.9±0.3 versus stress 1.8±0.5, *P*=0.666). However, in the nonobese group, *k*_f_^CK^ increased by 86±82% (*P*<0.001), and ATP delivery rate also increased (by 80±77%, *P*<0.001; Figure 3). However, in contrast, in the obese group despite similar hemodynamic changes to the control group (Table IV in the Data Supplement), no significant increase was observed in either *k*_f_^CK^ (rest 0.19±0.08 s^–1^ versus stress 0.18±0.08 s^–1^, *P*=0.117) or ATP delivery rate (rest 2.0±1.0 µmol·g^–1^·s^–1^ versus stress 1.9±1.4 µmol·g^–1^·s^–1^, *P*=0.608; Figure 3).

In addition, when nonobese and obese cohorts are combined, the change in *k*_f_^CK^ in response to dobutamine stress correlates with exercise capacity, either measured by peak Vo_2_ (*r* 0.426, *P*=0.013), or HR reserve (*r* 0.550, *P*=0.007, Table V in the Data Supplement and Figure II in the Data Supplement). This correlation is driven primarily by the nonobese group given the lack of increase in *k*_f_^CK^ in the obese cohort.

Overall, this suggests that during moderately increased workload, in contrast with the nonobese heart where ATP delivery rate and LV ejection fraction are seen to increase during catecholamine stress, the obese heart fails to increase myocardial ATP delivery through CK, and achieves a lesser degree of LV ejection fraction augmentation to stress.

### Impact of Weight Loss

Of the obese cohort (n=45) who underwent the weight loss intervention, 9 (20%) declined follow-up (Figure 1), and of the remaining 36, 27 (75%) lost weight (as defined by any reduction in body weight) over the intervention, with a mean weight loss of 11±5% and fat mass loss of 23±14% (Table 3). The weight of the remaining 9 of 36 participants did not change (+4±5%, *P*=0.113); there were no differentiating factors at baseline between the participants who did and did not lose weight (Tables VI and VII in the Data Supplement). The following results refer to only those 27 individuals who were successful in losing weight, unless stated otherwise.

**Table 3.**
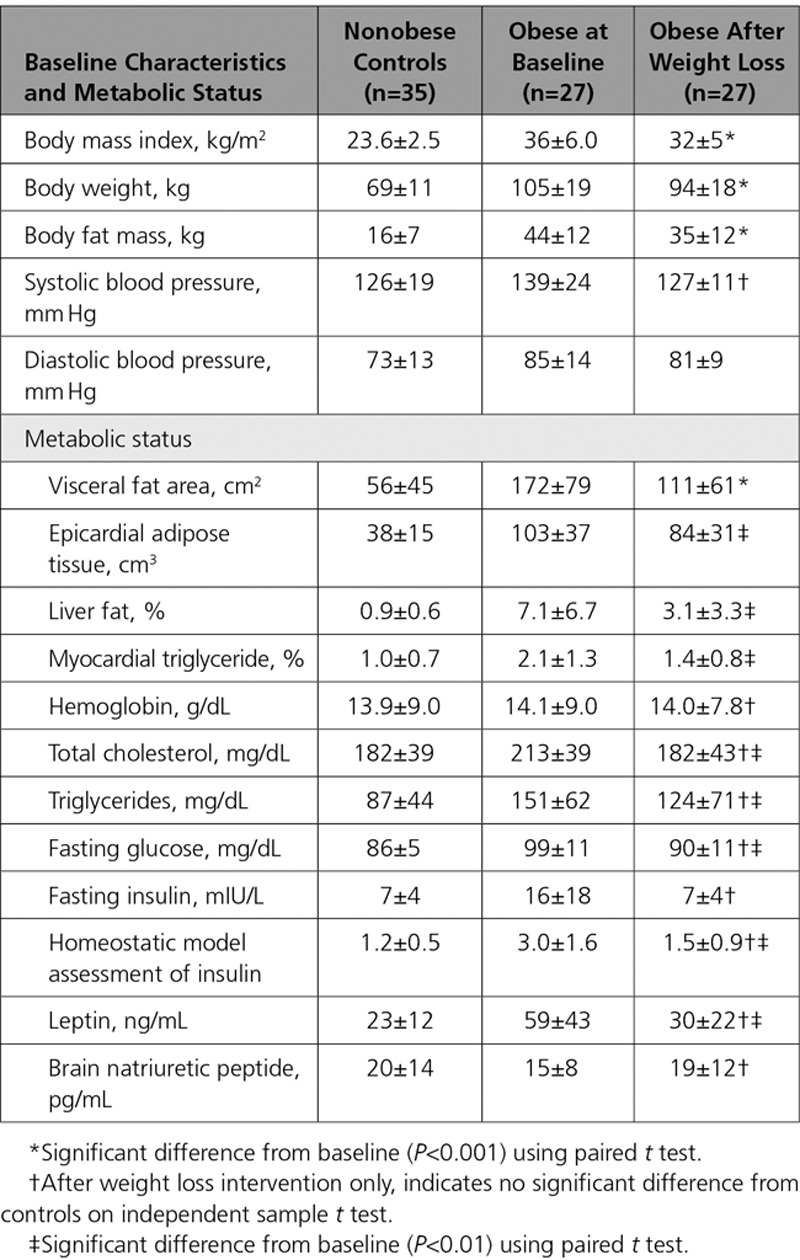
Anthropomorphic and Metabolic Consequences of Successful Weight Loss

Successful weight loss was associated with reduction in cholesterol (by 13±16%, *P*=0.001; Table 3), fasting glucose (by 9±11%, *P*=0.001), and homeostatic model assessment of insulin resistance (by 41±33%, *P*=0.003). Systolic blood pressure fell from 134±21 mm Hg to 126±12 mm Hg, albeit not reaching statistical significance with this sample size (*P*=0.085). Weight loss was associated with reduction in LV end-diastolic volume and mass, improvement in diastolic function, and maintenance of exercise capacity [Table 4, Figure III in the Data Supplement]), whereas no such changes were seen in those individuals who did not lose weight.

**Table 4.**
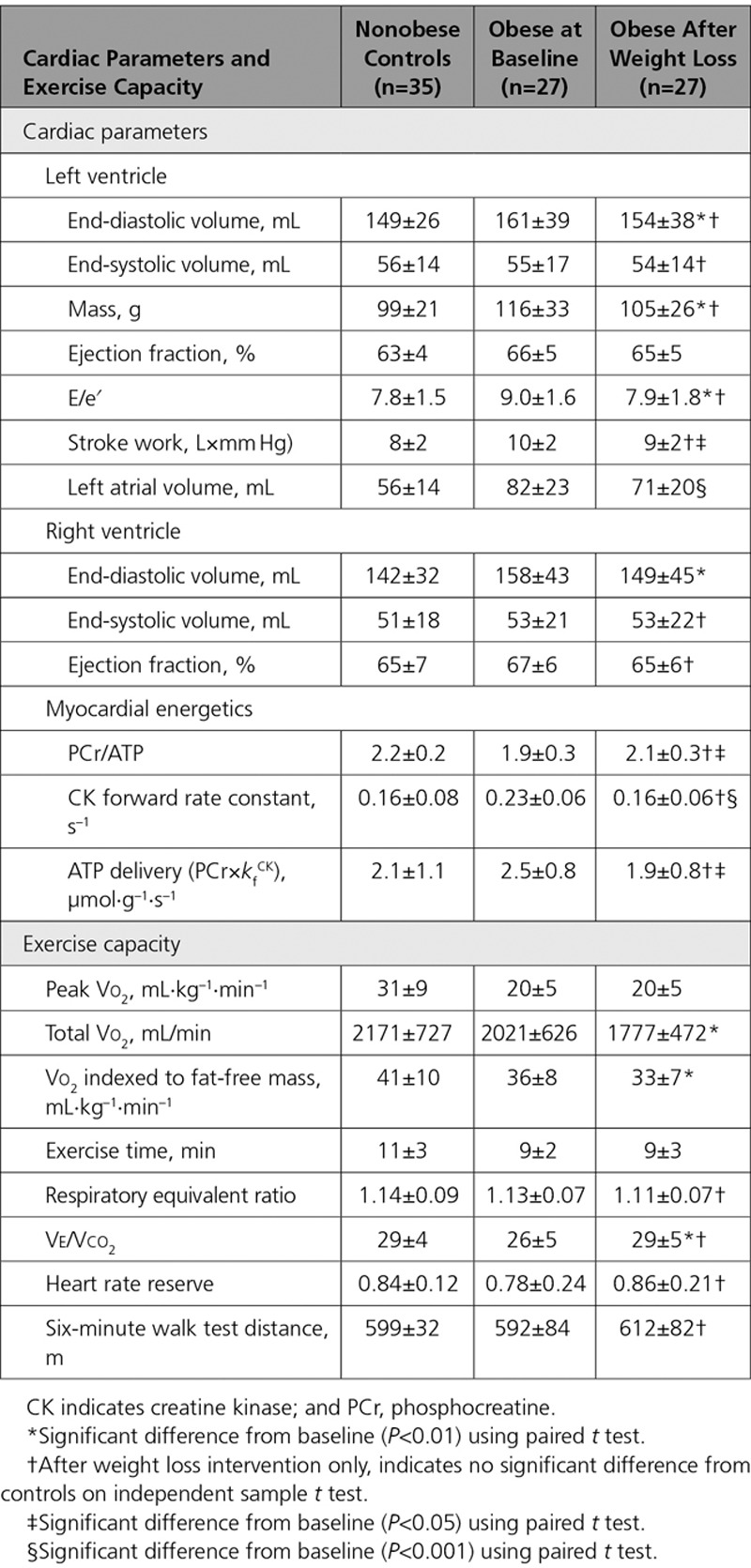
Impact on Cardiac Parameters and Exercise Capacity of Successful Weight Loss

#### Weight Loss and Myocardial Energetics

**Figure 4.**
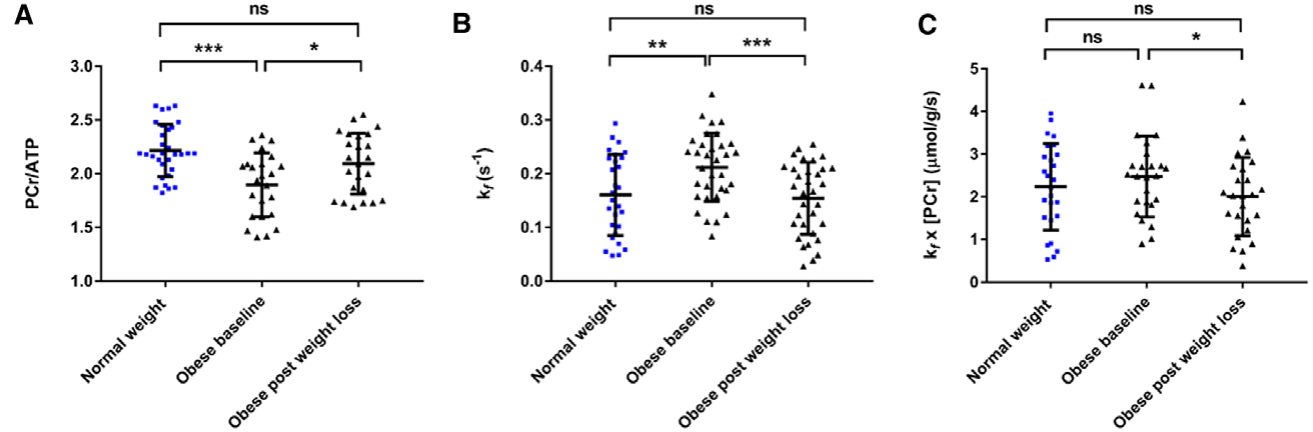
**The impact on myocardial energetics of weight loss from an obese baseline.** Weight loss in obesity led to restoration of PCr/ATP (**A**), creatine kinase forward rate constant (**B**), and ATP delivery (**C**). *P<0.05; **P<0.01; ***P<0.001 (within obese group change analyzed with paired *t* test; comparisons to control group with independent sample *t* test). ns indicates not significant; and PCr, phosphocreatine.

Weight loss was associated with an increase in PCr/ATP (from 1.9±0.3 to 2.1±0.3, *P*=0.040; Figure 4). Weight loss was also associated with a fall in both *k*_f_^Ckrest^ (from 0.23±0.06 s^–1^ to 0.16±0.06 s^–1^, *P*=0.001), and ATP delivery (from 2.5±0.8 µmol·g^–1^·s^–1^ to 1.9±0.8 µmol·g^–1^·s^–1^, *P*=0.018). After successful weight loss, all myocardial energetic parameters were statistically similar to those in the nonobese cohort (PCr/ATP control 2.2±0.2, obese after weight loss 2.1±0.3, *P*= 0.096; *k*_f_^Ckrest^ control 0.16±0.08 s^–1^, obese after weight loss 0.16±0.06 s^–1^, *P*=0.999; ATP delivery control 2.1±1.1 µmol·g^–1^·s^–1^, obese after weight loss 1.9±0.8 µmol·g^–1^·s^–1^, *P*=0.527). The change in *k*_f_^Ckrest^ correlated with weight change (*r*=0.397, *P*=0.045), change in visceral fat (*r*=0.485, *P*=0.019), and change in insulin (*r*=0.537, *P*=0.022) (Table V in the Data Supplement). Although the baseline correlation between *k*_f_^Ckrest^ and parameters of body composition is relatively weak, the relationship between change in *k*_f_ and change in fat supports an association between the 2 parameters.

This was in contrast with obese individuals who did not achieve weight loss, with no change in PCr/ATP (1.8±0.3 to 1.9±0.3 post, *P*=0.302, Table VII in the Data Supplement), *k*_f_^Ckrest^ (0.25±0.08 s^–1^ to 0.22±0.11 s^–1^ post, *P*=0.331), or ATP delivery (2.6±1.1 µmol·g^–1^·s^–1^ to 2.4±1.6 µmol·g^–1^·s^–1^, *P*=0.665).

#### Weight Loss and Stress Energetics

Of the original 17 obese individuals who underwent dobutamine stress energetics, only 6 completed repeat stress testing after a weight-loss intervention (of the others, 1 declined follow-up, 9 declined dobutamine, and 1 developed ectopy during infusion). Even with this small sample size, the obese heart after weight loss was apparently able to increase *k*_f_^CK^ (from 0.16±0.05 s^–1^ to 0.27±0.03 s^–1^, *P*=0.002) and ATP delivery (from 1.9±0.4 µmol·g^–1^·s^–1^ to 3.3±1.6 μmol·g^–1^·s^–1^, *P*=0.007) during increased workload (Figure IV in the Data Supplement). The absolute increase in LV ejection fraction with stress was now no longer statistically different from that achieved by nonobese controls (obese +16±5% versus nonobese +20±4%, *P*=0.088), although there is insufficient power to be confident that no difference exists.

Overall, this may suggest that, after successful weight loss, the obese heart at rest has statistically similar PCr/ATP, *k*_f_^Ckrest^, and ATP delivery rate to that seen in the nonobese heart, and that weight loss may lead to a restoration of the capacity to increase ATP delivery through CK during increased workload.

## Discussion

Obesity greatly increases the risk of developing heart failure with reduced ejection fraction,^3^ and is now one of the most common risk factors for heart failure with preserved ejection fraction, with recent studies identifying a distinct obesity-related heart failure with preserved ejection fraction phenotype.^18^ In addition, exertional intolerance is highly prevalent in obesity, limiting the feasibility of increasing physical activity as a therapeutic intervention, and having a dramatic impact on quality of life. However, the mechanisms underlying this are poorly understood.

**Figure 5.**
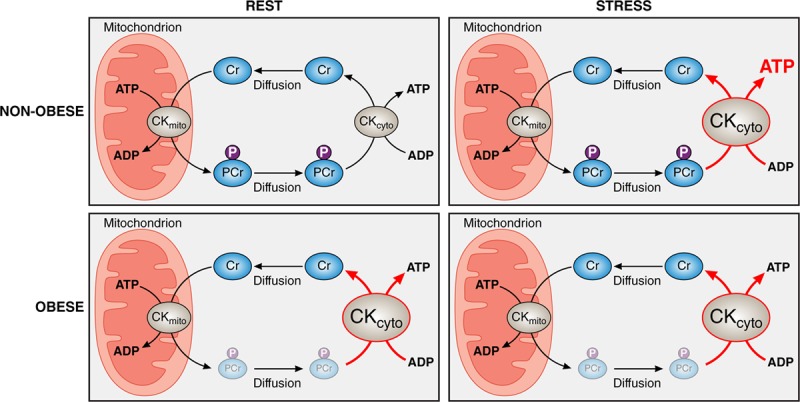
**Central illustration: the impact of obesity on myocardial ATP transfer through the creatine kinase system.** Creatine kinase facilitates rapid transfer of ATP from mitochondrion to myofibril by using rapid diffusion of phosphocreatine (**Top left**), enabling increased ATP delivery in response to demand (**Top right**). As body mass index increases, creatine kinase kinetics increase, compensating for falling PCr/ATP, and maintaining ATP delivery at rest (**Bottom left**); however, this increase comes at the cost of a blunted response to stress (**Bottom right**). CK_cyto_ indicates cytosolic creatine kinase; CK_mito_ indicates mitochondrial creatine kinase; Cr, creatine; P, phosphate group; and PCr, phosphocreatine.

Here, we show for the first time in humans that resting myocardial energy delivery is maintained despite low PCr/ATP, through a compensatory increase in CK kinetics. We also show that during stress, the nonobese heart increases its ATP delivery through CK; however, the obese heart is unable to do so (Figure 5). Last, we demonstrate that weight loss reverses these energetic changes. This highlights for the first time that, in obesity, while resting myocardial ATP delivery is maintained, the capacity of the stressed myocardium to augment ATP delivery and potentially contractile function is limited. This may be a potential energetic mechanism underlying both exercise intolerance in obesity, and also the propensity to develop heart failure.

### Cardiac Energetics at Rest

In obese individuals with normal global systolic function, the myocardial PCr/ATP ratio is reduced to a similar level seen in overt systolic failure, likely reflecting depletion of the creatine pool.^2^ Because a continual ATP supply to the myocardium is needed to maintain systolic function, it is reasonable to assume that there may be a compensatory mechanism at work, maintaining ATP delivery in the face of the diminished phosphocreatine pool. Given that ATP delivery through the CK shuttle is determined by the product of the pool size of phosphocreatine and the forward rate constant of the CK reaction, CK reaction kinetics are a likely candidate for this mechanism. Indeed, elevated CK reaction velocity has been shown in the isolated perfused hearts of obese rodents by using saturation transfer 31P MR spectroscopy^13,19^ and in ex vivo assays from human obese skeletal muscle.^20^ However, until recently, it was not possible to investigate this in the human heart in vivo. In this study, we demonstrate that, at rest, despite the reduced PCr/ATP ratio, myocardial ATP delivery through CK is maintained by a compensatory elevation in the forward rate constant of CK (*k*_f_^Ckrest^).

In contrast, previous in vivo human myocardial CK MR spectroscopy studies in different diseases, including dilated cardiomyopathy,^4^ hypertensive heart failure with reduced ejection fraction,^5^ and hypertrophic cardiomyopathy,^21^ have all demonstrated a reduced rate of CK kinetics in the direction of ATP generation. It is, however, important to note that these previous studies have focused on established disease processes. Because obesity is a strong risk factor for the development of heart failure, it is feasible that this compensatory increase in CK activity may preserve overall ATP delivery rate and systolic function in the early stages of the disease process, preceding eventual decompensation in both energetics and function. Thus, these adaptations may provide both an early marker of heart failure risk, and a potential therapeutic target to augment ATP delivery in cases of depleted substrate availability, given the difficulty in translating strategies to increase intracellular phosphocreatine.^22^

The molecular mechanisms underlying the elevated *k*_f_^Ckrest^ are as yet unknown. Possible candidate pathways could include changes in substrate affinity (isoform switch to B-CK), increases in CK total activity (increased gene expression), increased free [ADP] (this term is included within *k*_f_), and oxygen inefficiency.

Because β-adrenergic stimulation has been shown to alter the gene expression for CK, from M-CK to B-CK, which is characteristic for the hypertrophied and failing heart,^23^ and is present in obesity,^24^ it is plausible that this is a contributory factor. In addition, ATP supply and demand mismatch has also been shown to cause changes in CK isoenzyme expression. The accumulation of BB- or MB-CK allows subtle kinetic differences among CK isozymes to be exploited. On the basis of a higher affinity for ADP, the B-containing CK isozymes produce ATP more efficiently from PCr than the MM isoform. Thus, any increase in MB-CK could compensate for decreased energy reserve caused by decreased PCr content.

Last, because the obese heart may consume more oxygen per gram at rest for any given minute of work (because of increased futile proton cycling or increased relative reliance on fat oxidation versus carbohydrate oxidation), and oxygen consumption and ATP consumption and delivery are linearly correlated, the elevated *k*_f_ may simply reflect raised resting oxygen consumption. Furthermore, although we focus in this study on the likely spatial buffer capacity of the CK system, there are numerous potential additional roles^25^ that are outside the scope of this study.

### Response of the CK System to Increased Workload in Obesity

The pools of creatine and phosphocreatine, and the CK rate constant combine to produce the energy reserve for contraction. When cardiac workload is increased, cardiac demand for ATP increases and must be provided by both greater oxidative phosphorylation and accelerated ATP delivery. It has been demonstrated that ATP delivery through CK flux is associated with the degree of mechanical work performed by the heart.^26^ In this study, the elevated *k*_f_^Ckrest^ was proportional to the increased ventricular stroke work seen in obesity, consistent with the observation that it may be hemodynamic demand driving the resting enzymatic rate constant.

However, whereas in the nonobese heart both *k*_f_^CK^ and ATP delivery increase markedly during catecholamine-induced elevation in workload, in this study, no such increase is seen in obesity. The relative increase in ejection fraction is lower in obesity than controls; although it is interesting to note that there is an augmentation of contractile response despite no apparent increase in ATP delivery, suggesting that alternative pathways may be implicated.

Although the exact mechanisms involved remain to be elucidated, it is plausible that the compensatory increase in *k*_f_^Ckrest^ seen is maximal, preventing further augmentation during stress. In addition, ATP synthesis has also been shown to be reduced in obese rodents.^13^ The combination of reduced ATP synthesis and blunted *k*_f_^CK^ increase seen in this study would lead to energetic imbalance with increased free [ADP] during higher workload. This has been shown to cause a stepwise fall in the energy produced per molecule of ATP (ΔG_ATP_) as work is increased,^13^ eventually falling sufficiently to impact contractile reserve^13,27^ via sarcoendoplasmic reticulum Ca^2+^-ATPase dysfunction. Hence, although the elevation in *k*_f_^Ckrest^ observed here in humans may be sufficient to maintain ΔG_ATP_ to support the resting myocyte requirements, when the system is stressed, it is possible that these processes are no longer supported. Because adequate sarcoendoplasmic reticulum Ca^2+^-ATPase function is also critical to replenish calcium stores, a requirement for normal cardiac function, this would be consistent with the potentially limited systolic reserve observed in this study.

Although a potential explanation for this stress-induced limitation is microvascular dysfunction, known to be prevalent in this population,^28,29^ we feel that this is less likely in this particular cohort because of the lack of symptoms, regional wall motion abnormalities, or ECG changes with stress.

Given that exercise intolerance is so prevalent in obesity, these energetic changes are an attractive mechanism to explain fatigue and breathlessness, and potentially the decompensation in energetics in the trajectory toward heart failure, as well. This is particularly relevant to heart failure with preserved ejection fraction, where the cardinal feature of the disease process is a loss of the myocardial capacity to respond to stress^30^; it would be intriguing to investigate whether a similar energetic limitation is seen in heart failure with preserved ejection fraction, and whether this pathway could be a potential therapeutic target in a currently difficult-to-treat disease.

### Effects of Weight Loss on the CK System

The changes induced by weight loss on the wider cardiovascular system are well documented, with improvements in blood pressure, cardiac chamber size, cardiac hypertrophy, and both resting and exertional diastolic function all being reported.^31–33^ In addition, weight loss has previously been shown to improve myocardial PCr/ATP levels,^34^ which we confirm in this study. In addition, we have shown that, alongside this improvement in PCr/ATP at rest, there is a fall in *k*_f_^Ckrest^, accompanied by diastolic functional improvement. The reduction in *k*_f_^Ckrest^ with weight loss likely reflects the combination of increased phosphocreatine pool (as shown here by improved PCr/ATP), and reduced LV stroke work after weight loss. Despite improvements in myocardial energetics, diastolic function, and 6-minute walk test, weight loss was associated with a fall in total Vo_2_ and Vo_2_ indexed to fat-free mass, suggesting that the potential improvement in efficiency was outweighed by the fall in overall energy required by a smaller body mass.

In addition to this, we demonstrate that weight loss may improve the myocardial capacity to augment *k*_f_^CK^ and indeed contractile reserve with stress; however, this result would need reproducing in larger data sets for certainty given that the final numbers were underpowered here.

The partial restoration of PCr/ATP and resting *k*_f_^CK^, in combination with a possible return of capacity to increase ATP delivery with stress, are attractive mechanisms to explain the improved diastolic function and exercise tolerance seen with successful weight loss interventions. In addition, it underlines the importance of treating obesity and its physiological complications as a therapeutic intervention in the prevention and potential treatment of heart failure.

### Limitations

In the absence of invasive measurements, an established approximation of LV stroke work (mean arterial pressure×stroke volume was used, which may overestimate actual stroke work.

The measured value of *k*_f_^CK^ has been adjusted to allow for a difference in B0 variability between our participants who were studied supine, and previously published data usually acquired prone. This adjustment has been previously published^12^; however, it was derived from a normal weight population. Although it is valid to hypothesize that there would be no difference in B0 variability in obesity, this has not been validated in different patient populations.

There is some variation in the length of follow-up during the dietary intervention because of a combination of patient preference and technical issues; however, this is more reflective of a real-life intervention, and the volumetric and energetic changes associated with weight loss remain robust.

## Conclusion

Here, we demonstrate for the first time that obesity is associated with the maintenance of myocardial energy supply, via increased kinetics of the CK enzyme in human myocardium. However, this compensation comes at the expense of the capacity to increase ATP delivery during increased workload, alongside decreased systolic augmentation. Furthermore, we show that both the rest and stress changes observed are reversed by successful weight loss. This suggests, not only that obesity-induced cardiac dysfunction and exercise intolerance are at least partly related to energetic impairment, but that they are reversible with weight loss and therefore viable therapeutic targets to treat obesity cardiomyopathy.

## Sources of Funding

Dr Rodgers is funded by a Sir Henry Dale Fellowship from the Wellcome Trust and the Royal Society (098436/Z/12/B). Dr Rayner was funded by a British Heart Foundation Clinical Research Training Fellowship (FS/14/54/30946).

## Disclosures

None.

## Supplementary Material


