# Supplementary material for: Myocardial Energetics in Obesity: Enhanced ATP Delivery Through Creatine Kinase With Blunted Stress Response
Source: Circulation. 2020 Apr 6;141(14):1152–63. doi: 10.1161/CIRCULATIONAHA.119.042770 (PMC7144750; doi:10.1161/CIRCULATIONAHA.119.042770)

Supplemental Material

Supplemental Table 1: Correlation table demonstrating associations with PCr/ATP and creatine kinase kf:

	PCr/ATP		k_f^{CK} (s^{-1})	
	r	p	r	p
Age (y)	-0.242	**0.044**	-0.182	0.137
BMI (kg/m^2)	-0.437	**<0.001**	0.329	**0.006**
Body fat (% total)	-0.364	**0.009**	0.393	**0.006**
Abdominal visceral fat (cm^2)	-0.304	**0.021**	0.316	**0.020**
Epicardial adipose tissue (cm^3)	0.126	0.597	0.048	0.846
Myocardial triglyceride content (%)	-0.456	**0.002**	0.274	0.079
Fasting glucose (mg/dl)	-0.376	**0.006**	0.288	**0.043**
Fasting insulin (mIU/ml)	-0.067	0.638	0.188	0.196
HOMA- IR	-0.069	0.644	0.167	0.269
LV EDV (ml)	0.034	0.777	0.084	0.497
LVEF (%)	-0.234	0.052	0.140	0.255
LV E/e'	-0.117	0.462	0.117	0.726
LV stroke work (l/mmHg)	-0.089	0.495	0.309	**0.016**
PCr/ATP	-	-	-0.127	0.306
k_f^{CK} (s^{-1})	-0.127	0.306	-	-
BNP (pg/ml)	-0.035	0.798	-0.080	0.569
Peak VO_2 (m/kg/ min)	0.468	**<0.001**	-0.322	**0.020**
Absolute max VO_2 (ml/min)	0.309	**0.014**	-0.168	0.233

Supplemental Table 2: Comparison of baseline characteristics of those in the obese cohort who completed dobutamine stress and those who did not (anthropomorphic and metabolic findings)

	Completed dobutamine (n=17)	Did not complete dobutamine (n=28)	p
Age (y)	49±14	49±14	0.935
Female n (%)	11 (65)	22 (79)	0.393
BMI (kg/m^2)	36±5	35±4	0.265
Weight (kg)	103±18	99±14	0.367
Body fat mass (kg)	47±11	42±9	0.148
Heart rate (bpm)	65±9	67±15	0.537
Systolic blood pressure (mmHg)	134±15	135±19	0.919
Diastolic blood pressure (mmHg)	80±12	86±12	0.916
**Metabolic status:**			
Visceral fat area (cm^2)	143±60	160±88	0.531
Liver fat (%)	7±7	7±6	0.826
Myocardial triglyceride (%)	2.1±2.4	2.5±1.3	0.607
Haemoglobin (g/dL)	141±11	138±13	0.521
Total cholesterol (mg/dL)	201±43	217±35	0.196
Triglycerides (mg/dL)	66±31	70±35	0.765
Fasting glucose (mg/dL)	95±11	95±13	0.891
Fasting insulin (mIU/L)	14±8	15±16	0.865
HOMA-IR	3.6±2.4	3.9±5.0	0.908
BNP (pg/mL)	5.2±4.0	5.6±4.6	0.829
Leptin (ng/mL)	56±40	56±28	0.968

Supplemental Table 3: Comparison of baseline characteristics of those in the obese cohort who completed dobutamine stress and those who did not (cardiac and exercise parameters)

	Completed dobutamine (n=17)	Did not complete dobutamine (n=28)	p
**Cardiac parameters:**			
**Left ventricle:**			
End-diastolic volume (ml)	168±38	151±30	0.130
End-systolic volume (ml)	60±14	52±13	0.100
Cardiac output (l/min)	6.9±1.1	6.3±2.2	0.379
Mass (g)	119±32	110±27	0.299
Ejection fraction (%)	65±3	65±5	0.540
Mass-volume ratio	0.7±0.1	0.7±0.1	0.641
E/e’	9.3±2.8	8.9±1.8	0.593
Left atrial volume (mls)	85±31	75±14	0.260
**Right ventricle:**			
End-diastolic volume (ml)	162±41	144±36	0.165
End-systolic volume (ml)	54±18	47±16	0.185
Ejection fraction (%)	67±5	68±6	0.607
**Myocardial energetics:**			
PCr/ATP	1.9±0.3	1.9±0.3	0.701
CK forward rate constant (s^{-1})	0.22±0.07	0.25±0.05	0.199
ATP delivery (PCr x k_f^{CK}) (µmol/g/sec)	2.4±0.9	2.7±0.7	0.215
**Exercise capacity:**			
Peak VO_2 (ml/kg/min)	22±5	19±5	0.162
VO_2 indexed to fat-free mass (ml/kg/min)	39±7	34±7	**0.031**
Percentage of predicted VO_2 achieved (%)	89±26	87±14	0.816
Exercise time (min)	9±2	9±2	0.914
RER	1.1±0.1	1.1±0.1	0.915
VE/VCO_2	25±4	27±4	0.288
Heart rate reserve	0.8±0.1	0.8±0.2	0.805
Six minute walk test distance (m)	586±65	567±77	0.478

Supplemental Table 4: Haemodynamic response to stress

	**Non-obese n=27**	**Obese n=17**	**p**
Total duration dobutamine (mins)	24±3	25±2	0.676
Resting heart rate (bpm)	57±7	64±9	**0.012**
Stress heart rate (bpm)	113±8	114±10	0.858
Average heart rate (% maximum)	64±4	67±6	0.100
Resting systolic blood pressure (mmHg)	121±17	133±15	**0.029**
Maximum systolic blood pressure (mmHg)	151±14	168±19	0.005
Resting RPP (mmHg*bpm)	6899±1293	8591±1595	**0.001**
Stress RPP (mmHg*bpm)	17264±1793	19071±2085	**0.010**
Relative increase in RPP (%)	167±60	132±38	0.079
Resting LV ejection fraction (%)	62±5	66±4	**0.003**
Stress LV ejection fraction (%)	83±4	82±6	0.638
Relative increase in LV ejection fraction (%)	21±4	16±7	**0.031**
Relative increase in LV stroke work (%)	24±35	21±27	0.818

Supplemental Table 5: Correlation table of change in PCr/ATP and creatine kinase kf with weight loss and changes in other parameters

	Δ PCr/ATP		Δ k_f^{CK} (s^{-1})	
	r	p	r	p
Δ BMI (kg/m^2)	0.019	0.927	0.422	**0.032**
Δ Body fat (% total)	-0.041	0.847	0.412	**0.014**
Δ Myocardial triglyceride content (%)	0.008	0.978	0.099	0.644
Δ Fasting glucose (mg/dl)	-0.019	0.928	0.314	0.066
Δ Fasting insulin (mIU/ml)	-0.072	0.728	0.582	**<0.001**
Δ LV EDV (ml)	0.279	0.178	0.376	**0.009**
Δ LVEF (%)	0.090	0.804	0.342	0.139
Δ LV E/e'	0.430	0.052	-0.031	0.890
Δ BNP (pg/ml)	-0.022	0.930	-0.243	0.153
Δ Peak VO_2 (m/kg/ min)	0.183	0.439	-0.198	0.402
Δ Absolute max VO_2 (ml/min)	0.083	0.728	0.393	**0.015**

Supplemental Table 6: Impact of unsuccessful weight loss on anthropomorphic and metabolic parameters

	Successful weight loss n=27		Unsuccessful weight loss n=9	
	**Baseline**	**Follow-up**	**Baseline**	**Follow-up**
Age (y)	51±13	-	53±15	-
BMI (kg/m^2)	36±6.0	32±5***	35±5	36±4
Body weight (kg)	105±19	94±18***	97±19	101±23
Body fat mass (kg)	44±12	35±12***	44±12	44±14
Systolic blood pressure (mmHg)	139±24	127±11	143±17	141±13
Diastolic blood pressure (mmHg)	85±14	81±9	85±7	83±12
**Metabolic status:**				
Visceral fat area (cm^2)	172±79	111±61***	135±60	113±35
Epicardial adipose tissue (cm^3)	103±37	84±31**	-	-
Liver fat (%)	7.1±6.7	3.1±3.3**	6.4±9.3	3.8±3.5
Myocardial triglyceride (%)	2.1±1.3	1.4±0.8**	1.9±0.9	1.5±0.6
Haemoglobin (g/dL)	14.1±9.0	14.0±7.8	13.9±1.7	14.8±1.4
Total cholesterol (mg/dL)	213±39	182±43**	201±58	213±50
Triglycerides (mg/dL)	151±62	124±71**	142±106	133±71
Fasting glucose (mg/dL)	99±11	90±11**	93±27	88±14
Fasting insulin (mIU/L)	16±18	7±4	16±12	22±19
HOMA-IR	3.0±1.6	1.5±0.9**	4.6±3.3	6.2±4.9
Leptin (ng/mL)	59±43	30±22**	54±18	39±17*
BNP (pg/mL)	15±8	19±12	20±20	14±6

* indicates significant difference from baseline (* p<0.05, **p<0.01, ***p<0.001; # (unsuccessful weight loss group baseline data only) indicates significant difference from successful weight loss baseline)

Supplemental Table 7: Impact of unsuccessful weight loss on cardiac and exercise parameters

	Successful weight loss n=27		Unsuccessful weight loss n=9	
	**Baseline**	**Follow-up**	**Baseline**	**Follow-up**
**Cardiac parameters:**				
**Left ventricle:**				
End-diastolic volume (ml)	161±39	154±38**	156±17	145±16*
LVEDV indexed to BSA (ml/m^2)	73±12	74±13*	75±9	68±9***
End-systolic volume (ml)	55±17	54±14	57±8	56±14
LVESV indexed to BSA (ml/m^2)	24±6	26±5	27±2	27±10
Mass (g)	116±33	105±26**	115±29	110±30
LV mass indexed to BSA (g/m^2)	52±11	51±8	54±11	51±12
Ejection fraction (%)	66±5	65±5	64±2	67±4
E/e’	9.0±1.6	7.9±1.8**	10±4	10±3
Left atrial volume (mls)	82±23	71±20***	72±12	73±13
LA vol indexed to BSA (ml/m^2)	37±8	33±7**	34±7	34±7
**Right ventricle:**				
End-diastolic volume (ml)	158±43	149±45**	146±22	142±16
RVEDV indexed to BSA (ml/m^2)	72±14	71±17	69±10	66±4
End-systolic volume (ml)	53±21	53±22	48±12	48±9
RVESV indexed to BSA (ml/m^2)	24±7	25±8	23±4	22±4
Ejection fraction (%)	67±6	65±6	67±5	66±6
**Myocardial energetics:**				
PCr/ATP	1.9±0.3	2.1±0.3*	1.8±0.3	1.9±0.3
CK forward rate constant (s^{-1})	0.23±0.06	0.16±0.06***	0.25±0.08	0.22±0.11
ATP delivery (PCr x k_f^{CK}) (µmol/g/sec)	2.5±0.8	1.9±0.8*	2.6±1.1	2.4±1.6
**Exercise capacity:**				
Peak VO_2 (ml/kg/min)	20±5	20±5	19±4	19±3
Total VO_2 (ml/min)	2021±626	1777±472**	1767±379	1956±510*
VO_2 indexed to fat-free mass (ml/kg/min)	36±8	33±7**	34±4	33±2

Exercise time (min)	9±2	9±3	8±2	7±2
RER	1.13±0.07	1.11±0.07	1.10±0.12	1.10±0.04
VE/VCO_2	26±5	29±5**	26±5	26±5
Heart rate reserve	0.78±0.24	0.86±0.21	0.77±0.13	0.73±0.07
Six minute walk test distance (m)	592±84	612±82	576±38	552±22

* indicates significant difference from baseline (* $p<0.05$, ** $p<0.01$, *** $p<0.001$; # (unsuccessful weight loss group baseline data only) indicates significant difference from successful weight loss baseline)

Supplemental Figure 1: The contrasting effect of increasing visceral fat on PCr/ATP (A) and creatine kinase kinetics (B). Correlation coefficient displayed for combined groups.

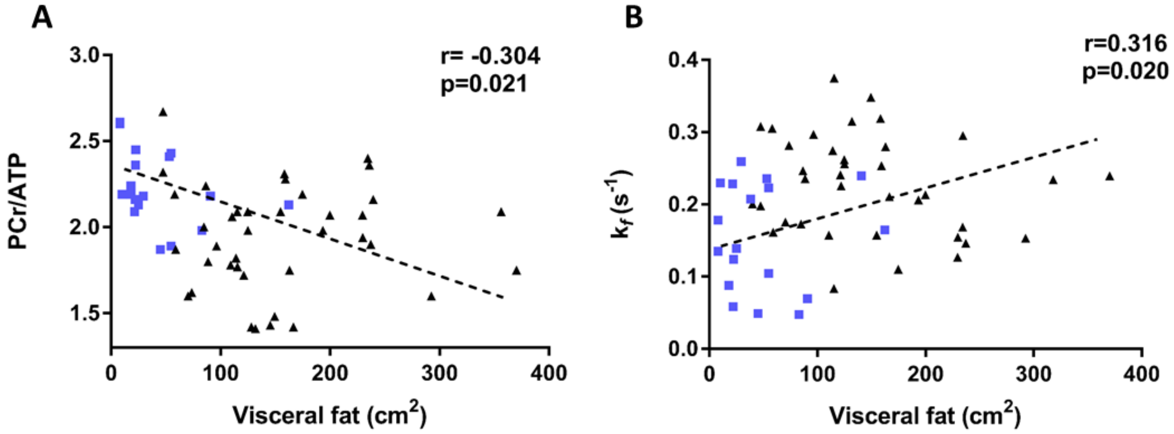

Supplemental Figure 2: The change in creatine kinase k_f with dobutamine stress correlates with both peak VO_2 (A) and heart rate reserve (B), when groups are combined.

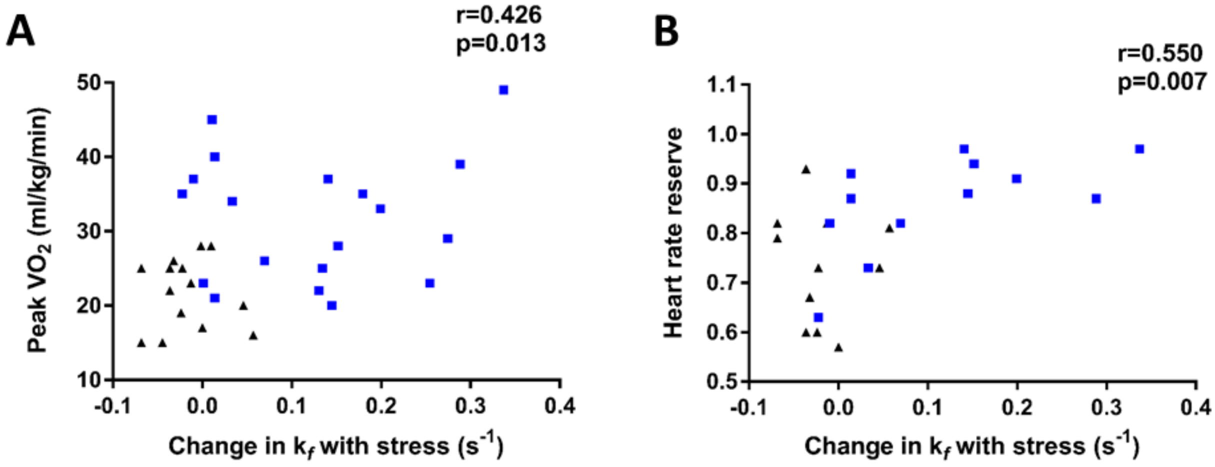

Supplemental Figure 3: Weight change correlates with change in six minute walk test distance (A) and exercise time (B)

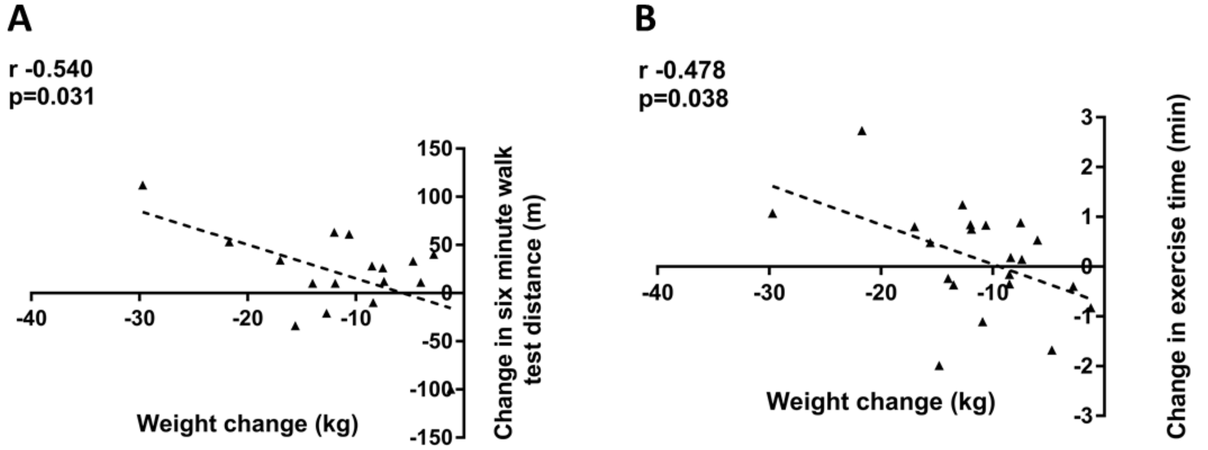

Supplemental Figure 4: Weight loss may lead to a greater stress response of creatine kinase k_f (comparison of creatine kinase kinetics before and after weight loss intervention in the same 6 individuals)

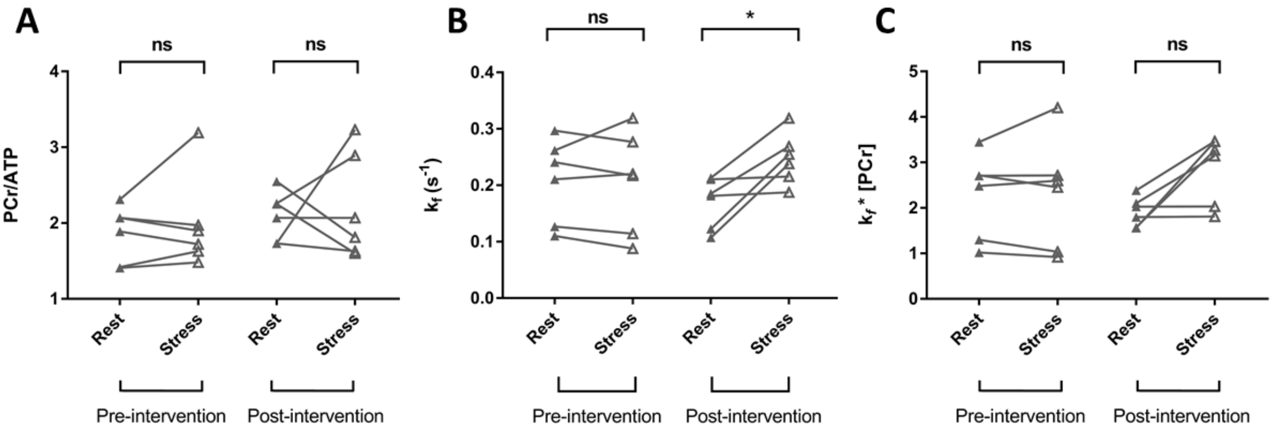

Supplement: Supplementary file 1 [file cir-141-1152-s001.pdf]
